# Postthrombotic syndrome and quality of life after deep vein thrombosis in patients treated with edoxaban versus warfarin

**DOI:** 10.1002/rth2.12748

**Published:** 2022-07-01

**Authors:** Ingrid M. Bistervels, Roisin Bavalia, Jan Beyer‐Westendorf, Arina J. ten Cate‐Hoek, Sebastian M. Schellong, Michael J. Kovacs, Nicolas Falvo, Karina Meijer, Dominique Stephan, Wim G. Boersma, Marije ten Wolde, Francis Couturaud, Peter Verhamme, Dominique Brisot, Susan R. Kahn, Waleed Ghanima, Karine Montaclair, Amanda Hugman, Patrick Carroll, Gilles Pernod, Olivier Sanchez, Emile Ferrari, Pierre‐Marie Roy, Marie‐Antoinette Sevestre‐Pietri, Simone Birocchi, Hilde S. Wik, Barbara A. Hutten, Michiel Coppens, Christiane Naue, Michael A. Grosso, Minggao Shi, Yong Lin, Isabelle Quéré, Saskia Middeldorp, the Hokusai PTS Investigators

**Affiliations:** ^1^ Department of Vascular Medicine, Amsterdam Cardiovascular Sciences, Amsterdam UMC University of Amsterdam Amsterdam The Netherlands; ^2^ Department of Internal Medicine Flevo Hospital Almere The Netherlands; ^3^ Department of Medicine I, Division of Hematology and Hemostasis, Thrombosis Research University Hospital "Carl Gustav Carus" Dresden Dresden Germany; ^4^ Thrombosis Expertise Centre, Heart+Vascular Center Maastricht University Medical Centre Maastricht The Netherlands; ^5^ Medizinische Klinik, Städtisches Klinikum Dresden Dresden Germany; ^6^ Department of Hematology and Thrombosis London Health Sciences Centre,Victoria Hospital London Ontario Canada; ^7^ Department of Internal Medicine and Immunology Centre Hospitalier Regionale Universitaire Dijon Dijon France; ^8^ Department of Hematology University Medical Centre Groningen Groningen The Netherlands; ^9^ Department of Hypertension, Vascular Disease and Clinical Pharmacology Regional University Hospital Strasbourg France; ^10^ Department of Pulmonology Noordwest Ziekenhuisgroep Alkmaar The Netherlands; ^11^ Department of Pulmonology Centre Hospitalier Regionale Universitaire Brest Brest France; ^12^ Department of Vascular Medicine and Hemostasis University Hospital Leuven Leuven Belgium; ^13^ Department of Vascular Medicine Clinique du Parc Castelnau le Lez France; ^14^ Department of Medicine McGill University Montreal Canada; ^15^ Department of Research, Østfold Hospital and Institute of Clinical Medicine University of Oslo Oslo Norway; ^16^ Department of Cardiology Centre Hospitalier Le Mans Le Mans France; ^17^ Department of Haematology St George Hospital Sydney New South Wales Australia; ^18^ Department of Vascular Medicine Redcliffe Hospital Queensland Australia; ^19^ Department of Medicine Centre Hospitalier Regionale Universitaire de Grenoble‐Alpes Grenoble France; ^20^ Department of Pulmonology Hôpital Européen Georges‐Pompidou Paris France; ^21^ Department of Cardiology Centre Hospitalier Universitaire de Nice Nice France; ^22^ Department of Emergency Medicine Centra Hospitalier Universitaire d'Angers Angers France; ^23^ Department of Medicine Centre Hospitalier Regionale Universitaire d'Amiens Amiens France; ^24^ Department of Hematology and Thrombosis SanPaolo Hospital Milan Italy; ^25^ Department of Haematology Oslo University Hospital Oslo Norway; ^26^ Department of Epidemiology and Data Science, Amsterdam Cardiovascular Sciences Amsterdam UMC, University of Amsterdam Amsterdam The Netherlands; ^27^ Daiichi Sankyo Pharma Development Basking Ridge New Jersey USA; ^28^ Department of Vascular Medicine IDESP Inserm‐Montpellier University, InnoVTE Network, CHU Montpellier Montpellier France; ^29^ Department of Internal Medicine & Radboud Institute of Health Sciences (RIHS)Radboud University Medical Center Nijmegen The Netherlands

**Keywords:** edoxaban, postthrombotic syndrome, quality of life, venous thrombosis, warfarin

## Abstract

**Background:**

Postthrombotic syndrome (PTS) is a long‐term complication after deep vein thrombosis (DVT) and can affect quality of life (QoL). Pathogenesis is not fully understood but inadequate anticoagulant therapy with vitamin K antagonists is a known risk factor for the development of PTS.

**Objectives:**

To compare the prevalence of PTS after acute DVT and the long‐term QoL following DVT between patients treated with edoxaban or warfarin.

**Methods:**

We performed a long‐term follow‐up study in a subset of patients with DVT who participated in the Hokusai‐VTE trial between 2010 and 2012 (NCT00986154). Primary outcome was the prevalence of PTS, defined by the Villalta score. The secondary outcome was QoL, assessed by validated disease‐specific (VEINES‐QOL) and generic health‐related (SF‐36) questionnaires.

**Results:**

Between 2017 and 2020, 316 patients were enrolled in 26 centers in eight countries, of which 168 (53%) patients had been assigned to edoxaban and 148 (47%) to warfarin during the Hokusai‐VTE trial. Clinical, demographic, and thrombus‐specific characteristics were comparable for both groups. Mean (SD) time since randomization in the Hokusai‐VTE trial was 7.0 (1.0) years. PTS was diagnosed in 85 (51%) patients treated with edoxaban and 62 (42%) patients treated with warfarin (adjusted odds ratio 1.6, 95% CI 1.0–2.6). Mean differences in QoL scores between treatment groups were not clinically relevant.

**Conclusion:**

Contrary to our hypothesis, the prevalence of PTS tended to be higher in patients treated with edoxaban compared with warfarin. No differences in QoL were observed. Further research is warranted to unravel the role of anticoagulant therapy on development of PTS.

**Essentials**

- Post‐thrombotic syndrome (PTS) is a long‐term complication of deep vein thrombosis (DVT).
- In this follow‐up study of the Hokusai‐VTE trial, we assessed PTS and quality of life (QoL).
- Seven years after index DVT, 316 patients were included in 26 centers in eight countries.
- PTS prevalence seemed higher in patients treated with edoxaban versus warfarin, QoL was similar.

## 1 INTRODUCTION

Postthrombotic syndrome (PTS) is a long‐term complication of deep vein thrombosis (DVT) that is reported by 20%–50% of patients.^1^ PTS is a chronic condition that includes a variety of signs and discomforting symptoms of the leg that persists after the acute phase of DVT. Patients with PTS can report chronic pain, (nocturnal) cramps, tingling, and heaviness of the leg. Furthermore, the appearance of the leg can be affected by swelling, hyperpigmentation, redness, and venous ectasia. Severity of signs and symptoms vary among patients and PTS is considered severe when venous insufficiency leads to ulcers and disability.^2^, ^3^ Generally, severe PTS comprises 5%–10% of the cases, but rates up to 22% have been reported.^4^, ^5^, ^6^ Although the pathogenesis is not completely understood, PTS is thought to be the result of impaired thrombus resolution; persistent venous obstruction could cause venous hypertension and valvular reflux leading to impaired microcirculation.^7^, ^8^, ^9^ The damage to the veins is irreversible in most cases. The burden of PTS has a significant impact on quality of life (QoL) and is associated with socioeconomic consequences.^3^, ^10^, ^11^, ^12^, ^13^ Self‐reported physical QoL scores of patients with severe PTS are comparable to scores of patients suffering from angina, cancer, or congestive heart failure.^14^

At present, therapeutic options are limited and, therefore, prevention of PTS is crucial. Studies on catheter‐directed thrombolysis failed to consistently show a reduction on PTS^15^, ^16^, ^17^ and evidence on the efficacy of elastic compression stockings (ECS) for PTS prevention is conflicting. Accordingly, current guidelines do not explicitly recommend the routine use of ECS.^18^, ^19^, ^20^, ^21^ Nevertheless, ECS may help to diminish PTS‐related symptoms, leading to an improved QoL. Given the relation of PTS to residual clot burden, preventive measures should focus on quality of anticoagulation.^22^, ^23^ Inadequate anticoagulant therapy with vitamin K antagonists (VKAs) (i.e., subtherapeutic international normalized ratio [INR]) is a known risk factor for the development of PTS.^24^, ^25^, ^26^, ^27^ In the past decade, VKAs have been replaced by direct oral anticoagulants (DOACs) as drug of first choice for most patients with an acute venous thromboembolism (VTE).^21^ DOACs are pharmacologically more stable than VKAs and do not require dose adjustments or intensive therapeutic drug monitoring. Hence, quality of anticoagulation may be higher for DOACs than with VKA, particularly in the days to weeks after discontinuation of the heparin lead‐in in patients with acute VTE. Several studies, both randomized and observational, suggested a moderate reduction of PTS associated with rivaroxaban,^28^, ^29^, ^30^, ^31^, ^32^, ^33^, ^34^, ^35^ but not with dabigatran.^36^

Edoxaban, a direct oral factor Xa inhibitor, is one of the DOACs that was proven as effective and safer with regard to serious bleeding in comparison with the VKA warfarin for treatment of acute VTE.^37^

In this study, we aim to assess the long‐term prevalence of PTS and health‐related quality of life in patients treated for DVT with edoxaban or warfarin. We hypothesize that patients treated with edoxaban have a lower prevalence of PTS and a better health‐related QoL compared with patients treated with warfarin for their index DVT.

## 2 METHODS

### 2.1 Study design and population

The source population for this cohort study consisted of the participants of the Hokusai‐VTE trial (NCT00986154).^37^ The Hokusai‐VTE trial was an international, randomized, double‐blind, noninferiority trial evaluating the efficacy and safety of edoxaban (30 or 60 mg daily) compared with warfarin (target INR between 2.0 and 3.0) in patients with acute symptomatic VTE. Between January 2010 and October 2012, 8292 patients were included, of which 5735 had DVT. All patients received initial therapy with subcutaneous enoxaparin for at least 5 days. Treatment with edoxaban or warfarin was continued for at least 3 months and for a maximum of 12 months. The last trial visit for all patients was scheduled 12 months after randomization.

The present Hokusai PTS study is part of the Hokusai post‐VTE study, which is dedicated to assessing long‐term outcomes (NCT04007653). We previously reported the methods and findings of the Hokusai post‐PE study.^38^ In short, in 2016, we approached 78 study centers, based on the number of patients included in the original Hokusai‐VTE trial (>10 patients) and language, and located in Austria, Australia, Belgium, Canada, Denmark, France, Germany, Italy, the Netherlands, New Zealand, Norway, United Kingdom, and United States of America to participate in the Hokusai post‐VTE study. Patients were eligible for the current follow‐up study if they were recruited in one of these study centers and treated for DVT (with or without PE) as their index event for participation in the Hokusai‐VTE trial. Patients were approached by their own study center: a letter with concise information about the Hokusai PTS study was sent to all patients who previously participated in the Hokusai‐VTE trial. Within weeks after receiving the study information, patients were called and invited for a single hospital visit, during which they underwent examination on the affected leg using the original Villalta score.^39^, ^40^ In addition, patients were asked to complete questionnaires on disease‐specific^41^, ^42^ and generic health‐related QoL.^43^, ^44^ Additional information on use of ECS, presence of residual thrombosis (in case imaging was performed), comorbidities, and medication use was collected. Data were entered in an international database platform (castor EDC database). Additionally, the original Hokusai‐VTE trial database was accessed and data on characteristics during trial inclusion (VTE history, thrombus characteristics, and management of DVT) as well as allocation of treatment were retrieved. In this database, compliance with edoxaban or matching placebo was analyzed as the percentage of doses taken of the planned number of doses during the study treatment period. Compliance with warfarin was analyzed by using the subjects' duration of time in the INR therapeutic range of 2.0–3.0 during the study treatment period.

The study protocol was approved by the institutional review board of Amsterdam UMC, University of Amsterdam (NL587525.018.16), and by local review boards in all participating centers. The study was registered at ClinicalTrials.gov (NCT04007653). The study was conducted according to the revised principles of the Declaration of Helsinki and in accordance with the Medical Research Involving Human Subjects Act; hence, all participants provided written informed consent.

An overview of the study timeline is presented in the Appendix S1, Figure S1.

### 2.2 Study outcomes

The primary outcome of this study was prevalence of PTS. The secondary outcomes included long‐term disease‐specific and generic health‐related QoL.

#### 2.2.1 Definition of postthrombotic syndrome

Diagnosis of PTS required assessment of the affected leg using the original Villalta scale.^39^, ^40^ This scale consists of 11 items that could be either absent (0 points), mild (1 point), moderate (2 points), or severe (3 points). Items cover both subjective symptoms reported by patients (heaviness, pain, cramps, itching, and tingling of the leg) and signs observed by trained assessors (pretibial edema, skin induration, hyperpigmentation, venous ectasia, redness, and pain on calf compression). The sum of the subjective and objective scores results in the Villalta score. The International Society on Thrombosis and Haemostasis consensus scoring method for PTS diagnosis was applied in this study: PTS was defined as a total Villalta score of 5 or higher or when a venous ulcer of the leg was present.^39^, ^45^ PTS severity was further classified as mild (Villalta score 5–9 points), moderate (Villalta score 10–14 points), or severe (Villalta score 15 or more or presence of venous ulcer).^39^, ^45^

#### 2.2.2 Quality of life assessment

The Venous Insufficiency Epidemiological and Economic Study‐ Quality of Life Questionnaire (VEINES‐QOL) is a validated questionnaire that measures the impact of DVT on symptoms and health‐related QoL in the past 4 weeks from the patient's perspective.^41^, ^42^ This questionnaire includes 26 items regarding leg symptoms, psychological impact, and limitations in daily life, of which 25 are used to calculate the VEINES‐QOL summary score. This score gives an overall estimation of venous disease‐specific QoL. The item that is not included in either score concerns intensity of symptoms over the day. Responses for each item are rated on a Likert response scale (2‐point to 7‐point), higher scores indicate better QoL. To be able to easily compare scores of different scales, raw scores were first transformed to *Z*‐score equivalents (mean, 0; standard deviation [SD], 1), and then transformed to T‐scores (mean, 50; SD, 10).^41^ We considered a mean difference of 4 points in the VEINES‐QOL T‐scores between the two treatment groups clinically relevant.^46^, ^47^

To evaluate the generic health‐related QoL regardless of any underlying disease, version 1 of the Short Form Health Survey questionnaire (SF‐36) was used.^43^, ^44^ This questionnaire comprises 36 items in which the general well‐being during the previous 30 days was assessed. It contains eight subscales: physical functioning, social functioning, physical role functioning, emotional role functioning, mental health, vitality, bodily pain, and general health. In addition to these eight domains, physical and mental summary scores were calculated. Scores are expressed on a 0–100 scale, with higher values indicating better general well‐being. We considered a mean difference of 10 points in the SF‐36 score between the two treatment groups clinically relevant.^48^

### 2.3 Statistical analysis

Statistical analysis was conducted according to the statistical analysis plan for the Hokusai post‐VTE studies and therefore similar to the Hokusai post‐PE study.^38^ Differences in baseline characteristics between both groups were evaluated by means of the 2‐sample *t* tests for normally distributed variables and the Mann–Whitney test for skewed distributions; χ^2^ tests were applied for categorical data.

The association between the occurrence of PTS (yes/no) and treatment group (edoxaban, warfarin) was assessed using logistic regression analysis. We evaluated the association between QoL outcomes (VEINES‐QOL and SF‐36) and treatment group (edoxaban, warfarin) by means of linear regression analysis, per subscale separately. We explored the effect of potential confounders in two different ways. First, potential confounders were assigned by clinical relevance by multiple assessors (R.B., I.B., B.H.) and were taken into a full model. Variables included in the full clinical model (model 1) for PTS outcomes were age, sex, body mass index (BMI), thrombotic history, cardiovascular disease, concomitant medication, awareness of randomized treatment, thrombus location of index DVT, and duration of anticoagulant treatment. For QoL outcomes, the full clinical model (model 1) included age, sex, BMI, thrombotic history, cardiovascular disease, musculoskeletal disease, neurological disease, psychiatric disorder, chronic analgesic use, thrombus location of index DVT, compliance to assigned treatment, and awareness of randomized treatment. Second, we determined the univariable association between patient characteristics (potential confounders) and treatment status (determinant), as well as between patient characteristics and outcome variables. Those characteristics that had a *p* value below 0.25 for both univariable associations were considered as a potential confounder and were included in a second full model (model 2). For both full models, we applied stepwise backward elimination based on the largest *p* value and created a final model (that always contained the variable ‘treatment allocation’), consisting of variables with a *p* value below 0.1.

We performed a sensitivity analysis in the subgroups of patients who were unaware of treatment allocation during the Hokusai‐VTE trial when participating in the Hokusai PTS study, and patients who stopped versus continued using anticoagulants after discontinuation of the Hokusai‐VTE trial until inclusion in the Hokusai PTS study.

### 2.4 Data sharing statements

For original data, please contact i.m.bistervels@amsterdamumc.nl.

## 3 RESULTS

**FIGURE 1:**
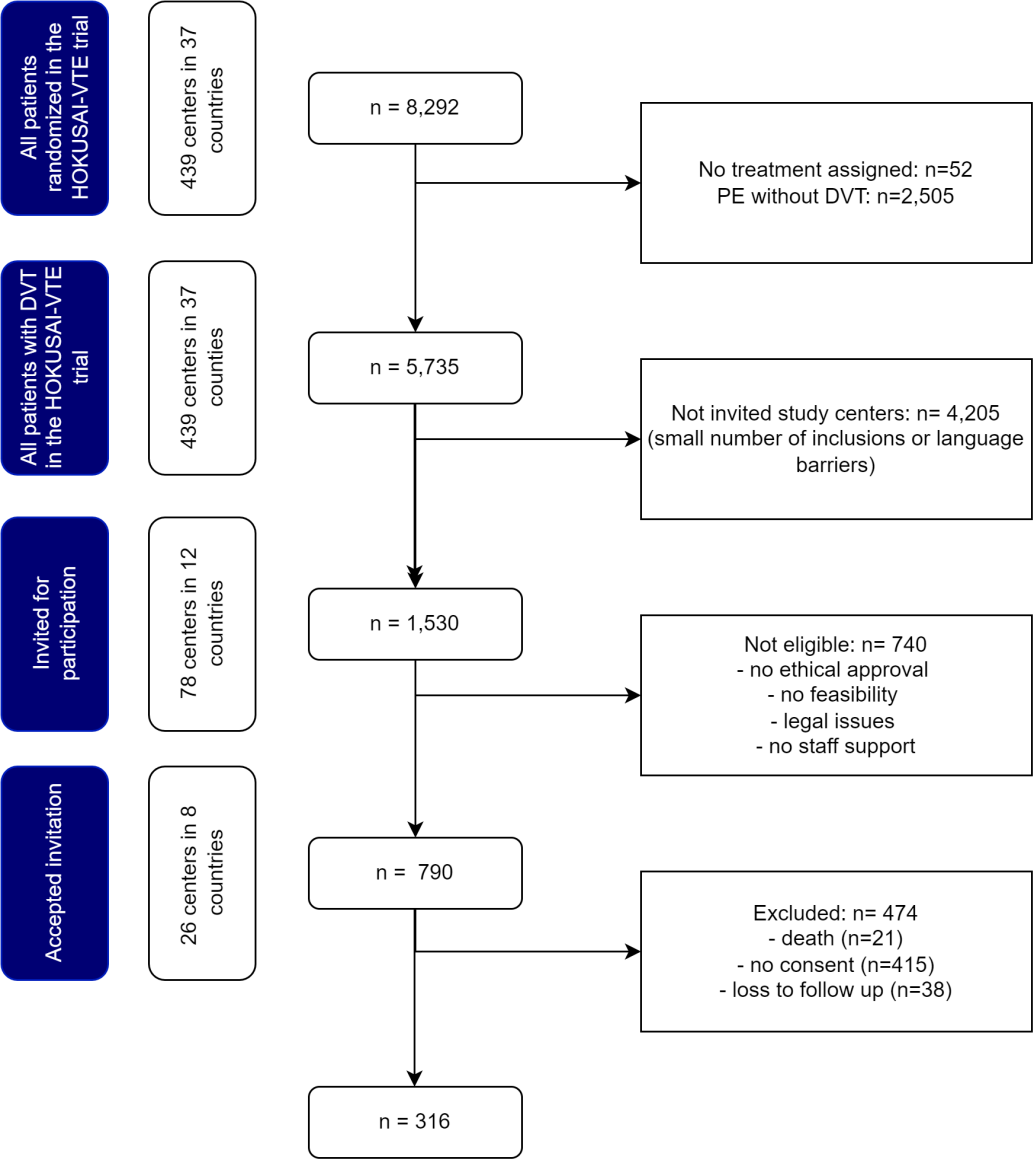
Flow chart patient selection Hokusai PTS study

Between April 2017 and September 2020, 316 patients were included in the Hokusai PTS study. Figure 1 represents the flowchart of patient inclusions in this study. The Hokusai‐VTE trial included 8292 patients in 439 centers in 37 countries. Of these randomized patients, 5735 were diagnosed with a DVT and used at least one dose of assigned study treatment.^37^ During site selection for the current study, 78 centers (1530 Hokusai‐VTE trial patients) were approached. Of these centers, 52 centers (740 Hokusai‐VTE trial patients) were not eligible for participation for logistical reasons (no ethical approval obtained, unable to approach participants, no study staff available). In the remaining centers, 474 of potentially eligibly 790 patients were excluded due to refusal of or inability to ask for consent for participation (*n* = 415), lost to follow‐up (*n* = 38), or death (*n* = 21), leaving 316 (5.5%) patients from 26 centers in eight countries for inclusion. The included countries were Australia, Belgium, Canada, France, Germany, Italy, Norway, and the Netherlands.

### 3.1 Patients and treatment

The mean time (SD) from randomization in the Hokusai‐VTE trial to inclusion in the Hokusai PTS study was 7 (1) years. Demographic and clinical characteristics at inclusion of the Hokusai PTS study are summarized in Table 1. The mean age (SD) at time of inclusion in the present study was 64 (14) years, mean BMI was 29 (6) kg/m^2^, 142 (45%) patients had radiologically confirmed residual thrombosis, and 78 (25%) had a recurrent VTE in their medical history.

**TABLE 1:** Demographic and clinical characteristics at inclusion of the Hokusai PTS study

Baseline characteristics	Total (*n* = 316)	Edoxaban^a^ (*n* = 168)	Warfarin^a^ (*n* = 148)
Mean age in years (SD)	63.5 (14.0)	64.0 (14.1)	62.7 (14.3)
Male sex, *n* (%)	171 (54.1)	101 (60.1)	70 (47.3)
Mean weight in kg (SD)	85.4 (19.7)	86.1 (20.7)	84.9 (18.3)
Mean BMI in kg/m^2^ (SD)	28.5 (5.5)	28.4 (5.3)	28.6 (5.8)
Smoking, *n* (%)	39 (12.4)	18 (10.7)	21 (14.3)
Residual thrombosis^b^, *n* (%)			
Yes	142 (45.1)	76 (45.5)	66 (44.6)
Unknown	57 (18.1)	34 (20.4)	23 (15.5)
≥2 VTE in history, *n* (%)	78 (24.7)	43 (25.6)	35 (23.6)
Comorbidities, *n* (%)			
Cardiovascular disease	172 (54.4)	98 (58.3)	74 (50.0)
Malignancy	30 (9.6)	16 (9.6)	14 (9.5)
Musculoskeletal disease	63 (19.9)	33 (19.6)	30 (20.3)
Neurological disease	17 (5.4)	12 (7.2)	5 (3.4)
Psychiatric disorder	17 (5.4)	5 (3.0)	12 (8.1)
Concomitant medication use, *n* (%)			
Any concomitant medication	247 (78.4)	132 (79.0)	115 (77.7)
Chronic analgesic use^c^	54 (17.1)	22 (13.1)	32 (21.6)
Chronic anticoagulant use	127 (40.2)	65 (38.7)	62 (41.9)
DOAC	73 (57.5)	37 (56.9)	36 (58.1)
VKA	52 (40.9)	26 (40.0)	26 (41.9)
Other	2 (1.6)	2 (3.1)	0 (0)
Years since randomization in Hokusai‐VTE trial, mean (SD)	7.1 (1.0)	7.0 (1.0)	7.2 (1.0)
Informed about treatment allocation during Hokusai‐VTE trial, *n* (%)	99 (31.3)	53 (31.5)	46 (31.1)

*Note*: Missing values: missing for age: 8, missing for weight: 6, missing for BMI: 8, missing for smoking: 1.

Abbreviations: BMI, body mass index; DOAC, direct oral anticoagulant; SD, standard deviation, VKA, vitamin K antagonists; VTE, venous thromboembolism.

^a^ Both edoxaban and warfarin treatment were preceded by enoxaparin.

^b^ Residual thrombosis was not predefined in the study protocol, investigators scored “present” when a residual thrombosis was reported by radiologists following the hospital’s definition.

^c^ Chronic analgesic use as reported by patients.

Of the 316 patients, 168 (53%) had been allocated to edoxaban and 148 (47%) to warfarin in the original Hokusai‐VTE trial. There was a higher proportion of men in patients treated with edoxaban in comparison with warfarin (60% vs. 47%; *p* = 0.02). The percentage of patients with a comorbidity was similar in the treatment groups, whereas the proportion of patients on chronic analgesics was lower in patients treated with edoxaban (13% vs. 22%; *p* = 0.05; Table 1).

Specifications on thrombus location and management of DVT during the Hokusai‐VTE trial were comparable in patients treated with edoxaban and warfarin (Table 2). The most proximal thrombus location was the popliteal vein in 124 (39%) patients, the superficial femoral vein in 113 (36%) patients and the common femoral or the iliac vein in 74 (23%) patients. Patients were treated for a median duration of 8 months (interquartile range [IQR] 6–12). All of the edoxaban‐treated and almost all of the warfarin‐treated (99%) patients were compliant to therapy ≥80% of the time). Twenty‐three patients (14%) of those treated with edoxaban, received an adjusted dose of edoxaban (30 mg once daily). In the warfarin group, the mean (SD) percentage of time in the therapeutic INR range was 70% (16%) and the mean (SD) percentage of time INR <2.0 was 13% (12%). Elastic compression stockings were used in 257 (81%) of patients. The majority (62%) of patients had worn the stockings for more than 2 years. Duration and use of ECS were comparable for both treatment groups.

**TABLE 2:** Characteristics of index DVT at randomization of Hokusai‐VTE trial

	Total (*n* = 316)	Edoxaban^a^ (*n* = 168)	Warfarin^a^ (*n* = 148)
Specific characteristics of DVT			
Thrombus location (most proximal site), *n* (%)			
Popliteal vein	124 (39.2)	63 (37.5)	61 (41.2)
Superficial femoral vein	113 (35.8)	59 (35.1)	54 (36.5)
Common femoral or iliac vein	74 (23.4)	42 (25.0)	32 (21.6)
Unknown	5 (1.6)	4 (2.4)	1 (0.7)
Unprovoked DVT, *n* (%)	189 (59.8)	99 (58.9)	90 (60.8)
Concomitant PE, *n* (%)	45 (14.2)	21 (12.5)	24 (16.2)
Treatment of DVT			
Median duration of anticoagulant treatment in months (IQR)	8.0 (6.0–12.0)	7.1 (6.0–12.0)	8.8 (6.0–12.0)
≥80% compliance to assigned treatment^b^, *n* (%)	314 (99.7)	168 (100.0)	146 (99.3)
Patients receiving 30 mg of Edoxaban at randomization^c^, *n* (%)	NA	23 (13.7)	NA
Mean percentage of time in therapeutic range^d^ (SD)	NA	NA	70.2 (15.7)
Mean percentage of time INR <2 (SD)	NA	NA	12.9 (11.5)
Use of concomitant medication, *n* (%)			
Antiplatelet treatment	13 (4.1)	10 (5.9)	3 (2.0)
NSAIDs	66 (20.9)	32(19.0)	34 (23.0)
Elastic compression stocking use, *n* (%)	257 (81.3)	138 (82.1)	119 (80.4)
<1 year	47 (18.3)	23 (16.7)	24 (20.2)
1–2 years	52 (20.2)	31 (22.5)	21 (17.6)
>2 years	158 (61.5)	84 (60.9)	74 (62.2)

Abbreviations: DVT, deep vein thrombosis; INR, international normalized ratio; IQR, interquartile range; NA, not applicable; NSAIDs, nonsteroidal anti‐inflammatory drugs; PE, pulmonary embolism; SD, standard deviation.

^a^ Both edoxaban and warfarin treatment were preceded by enoxaparin.

^b^ Compliance with edoxaban or matching placebo was analyzed as the percentage of doses taken of the planned number of doses during the study treatment period. Compliance with warfarin was analyzed by using the subjects' duration of time in the INR therapeutic range of 2.0–3.0 during the study treatment period.

^c^ Patients with a body weight below 60 kg or a creatinine clearance of 30–50 ml per minute, as well as patients who were receiving concomitant P‐glycoprotein inhibitors such as verapamil or quinidine, received 30 mg instead of 60 mg of edoxaban to maintain similar exposure to the cohort receiving 60 mg.

^d^ Time in therapeutic range is defined as percentage of time INR was between 2.0 and 3.0.

Following unblinding at the end of Hokusai‐VTE trial in some centers, at the time of participation in the Hokusai PTS study, 99 (31%) patients were aware of treatment allocation during the Hokusai‐VTE trial. Of 168 patients allocated to edoxaban, 85 (51%) patients stopped and 65 (39%) patients continued using anticoagulants after discontinuation of the Hokusai‐VTE trial until inclusion in the Hokusai PTS study. Of 148 patients allocated to warfarin, 76 (51%) stopped and 62 (42%) continued anticoagulant use.

Appendix S1 shows the corresponding baseline characteristics for all patients with DVT included in the Hokusai‐VTE trial, for patients from study sites that were not invited for the participation in the Hokusai PTS study, and for patients from study sites that were invited for participation (Appendix S1, Table S1). In the present study, the overall proportion of patients with thrombosis affecting the common femoral or iliac vein was almost half of the proportion in the Hokusai‐VTE trial (23% vs. 40%, respectively). Moreover, the duration of treatment was shorter in patients from sites invited for participation in the Hokusai PTS study. Last, the body weight and proportion of patients using edoxaban 30 mg is higher for patients from study sites invited for participation compared with those from sites not invited for participation.

### 3.2 Post‐thrombotic syndrome

PTS was diagnosed in 85 (51%) patients treated with edoxaban and in 62 (42%) patients treated with warfarin (crude odds ratio [OR] 1.4, 95% confidence interval [CI] 0.9–2.2; Table 3). After adjustment for all variables in the final regression models, the adjusted OR was 1.6 (95% CI 1.02–2.6) for model 1 (based on clinical reasoning) and 1.6 (95% CI 0.97–2.6) for model 2 (based on *p* values). The included variables in the adjusted models are presented in detail in the Appendix S1 (Table S1). Among patients with PTS treated with edoxaban, the disease was mild in 72%, moderate in 18%, and severe in 11%. Among patients treated with warfarin, the disease was mild in 65%, moderate in 26%, and severe in 10%. Ipsilateral leg ulcer was present in six (4%) patients treated with edoxaban and in one (1%) patient treated with warfarin (crude OR 5.4 [95% CI 0.6–45.7], adjusted OR 4.5 [95% CI 0.5–39.0, model 1], and 5.3 [95% CI 0.6–45.2, model 2]; Table 3).

**TABLE 3:** Study outcomes Hokusai PTS study

Outcome					
Primary outcome, *n* (%)	Edoxaban^a^ (*n* = 168)	Warfarin^a^ (*n* = 148)	Crude OR (95% CI)	Adjusted OR (95% CI)^c^	Adjusted OR (95% CI)^d^
Postthrombotic syndrome according to International Society on Thrombosis and Haemostasis scoring^b^	85 (50.9)	62 (42.2)	1.4 (0.9–2.2)	1.6 (1.02–2.6)	1.6 (0.97–2.6)
Villalta severity score					
Mild PTS (5–9)	61(71.8)	40 (64.5)	–	–	–
Moderate PTS (10–14)	15 (17.6)	16 (25.8)	–	–	–
Severe PTS (>14 or leg ulcer)	9 (10.6)	6 (9.7)	1.3 (0.5–3.9)	1.3 (0.4–3.9)	1.5 (0.5–4.8)
Ipsilateral leg ulcer	6 (3.6)	1 (0.7)	5.4 (0.6–45.7)	4.5 (0.5–39.0)	5.3 (0.6–45.2)

Secondary outcome, mean (SD)	Edoxaban^a^ (*n* = 168)	Warfarin^a^ (*n* = 148)	Crude mean difference (95% CI)	Adjusted mean difference (95% CI) ^c^	Adjusted mean difference (95% CI) ^d^
VEINES‐QOL	50.2 (9.6)	49.7 (10.4)	0.5 (−1.8 to 2.7)	−0.9 (−3.0 to 1.2)	−0.4 (−2.4 to 1.6)
SF‐36, PCS	45.7 (10.6)	46.6 (10.2)	−0.9 (−3.2 to 1.5)	−2.2 (−4.4 to 0.04)	−1.9 (−3.9 to 0.09)
SF‐36, MCS	52.6 (9.7)	51.5 (9.9)	1.1 (−1.1 to 3.3)	−0.1 (−2.2 to 2.1)	1.0 (−1.2 to 3.2)

*Note*: Missing values: Villalta score was not assessed in two patients (one treated with edoxaban, one treated with warfarin, VEINES‐QOL was missing for four patients (two treated with edoxaban, two treated with warfarin), SF‐36 PCS, and MCS was missing for 13 patients (six treated with edoxaban, seven treated with warfarin).

Abbreviations: 95% CI, 95% confidence interval; MCS, mental component score; OR, odds ratio; PCS, physical component score; PTS, postthrombotic syndrome; SF‐36, Short Form 36 items; VEINES‐QOL, Venous Insufficiency Epidemiological and Economic Study‐Quality of Life Questionnaire.

^a^ Both edoxaban and warfarin treatment were preceded by enoxaparin.

^b^ Villalta score of 5 or higher or presence of venous ulcer, at least 6 months after DVT.

^c^ Adjusted by variables from model 1 (clinical reasoning) as described in the methods section, model 1 included age, sex, body mass index, thrombotic history, cardiovascular disease, concomitant medication use, awareness of randomized treatment, thrombus location, and duration of anticoagulant use. Details on the included variables per model are presented in the Appendix S1.

^d^ Adjusted by variables derived by model 2 (*p* value <0.25) as described in the methods section, model 2 included sex, cardiovascular disease, chronic analgesic use, and concomitant antiplatelet therapy. Details on the included variables per model are presented in the Appendix S1.

### 3.3 Quality of life

Mean (SD) T‐score for VEINES‐QOL was 50.2 (9.6) for patients treated with edoxaban and 49.7 (10.4) for patients treated with warfarin (crude mean difference 0.5, 95% CI −1.8 to 2.7) (Table 3). After adjustment for possible confounders in the adjusted models, the mean differences in VEINES‐QOL score remained not significant and below 1 point.

The crude mean Physical Component Score of SF‐36 was 45.7 (SD 10.6) for patients treated with edoxaban and 46.6 (10.2) for patients treated with warfarin (−0.9, 95% CI −3.2 to 1.7). The adjusted mean difference for Physical Component Score was −2.2 (95% CI −4.4 to −0.04). The crude mean Mental Component Score was 52.6 (SD 9.7) for patients treated with edoxaban and 51.5 (SD 9.9) for patients treated with warfarin (1.1, 95% CI −1.1 to 3.3; Table 3). The adjusted mean Mental Component Score difference was comparable.

**FIGURE 2:**
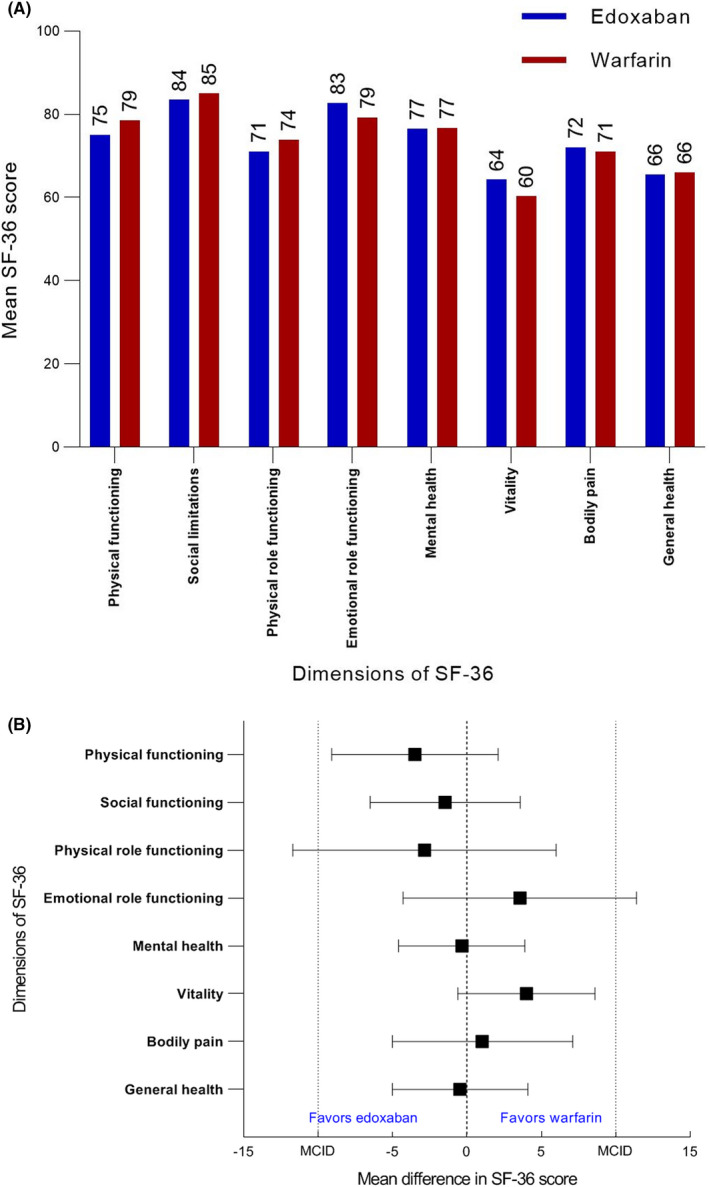
(A) Generic health‐related quality of life according to treatment of acute DVT. Mean SF‐36 scores of patients with a history of acute deep vein thrombosis in patients treated with edoxaban (*n* = 168) and warfarin (*n* = 148). This graph is not corrected for any baseline characteristics. Abbreviations for SF‐36 domains: GH, general health; ME, mental health; P, pain; PF, physical functioning; RE, role emotional complaints; RP, role physical complaints; SF, social functioning; VI, vitality. Missing values: missing physical functioning: 9, missing social functioning: 8, missing role physical complaints: 12, missing role emotional: 10, missing mental: 4, missing vitality: 8, missing pain: 8, missing general health: 9. (B) Mean difference in generic health‐related quality of life according to treatment of acute DVT. Mean difference and 95% confidence interval of SF‐36 scores of patients with a history of deep vein thrombosis in patients on edoxaban (n = 168) and warfarin (n = 148), stratified per dimension. This graph is not corrected for any baseline characteristics. MCID is the minimal clinically important difference, as described in the methods section. Abbreviations for SF‐36 domains: BP, bodily pain; GH, general health; ME, mental health; PF, physical functioning; RE, role emotional complaints; RP, role physical complaints; SF, social functioning; VI, vitality

In Figure 2A, the scores of the eight dimensions of the SF‐36 are displayed for patients treated with edoxaban and patients treated with warfarin. Crude mean differences were smaller than the minimal clinically important difference for all domains and were not statistically significant (Figure 2B). The adjusted mean difference for the physical functioning domain was −5.6 and statistically significant (95% CI −10.7 to −0.6). A detailed overview of the crude and adjusted differences in SF‐36 QoL domains are depicted in the Appendix S1 (Table S1; Table S1 for included variables in the adjusted models).

Sensitivity analyses in patients who were unaware of the allocation of treatment during the Hokusai‐VTE trial (*n* = 217) (Appendix S1, Table S1), and in patients who stopped (*n* = 161) and who continued (*n* = 127) using anticoagulants after discontinuation of the Hokusai‐VTE trial until inclusion in the Hokusai PTS study (Appendix S1, Table S1), did not show any large differences in comparison to the analysis in all 316 patients.

## 4 DISCUSSION

Contrary to our hypothesis, we observed that 7 years after enrollment in the Hokusai‐VTE trial patients with acute, symptomatic DVT who had been treated with edoxaban tended to have a higher risk for PTS compared with patients treated with warfarin. This was driven by a larger proportion of mild and moderate PTS because there was no difference in the prevalence of severe PTS between both treatment groups. No clinically relevant differences were observed in long‐term disease‐specific and generic health‐related QoL between treatment groups.

This is the first study to evaluate the PTS prevalence in patients treated with edoxaban versus warfarin. In two similarly designed studies (i.e., follow‐up of patients who previously participated in randomized controlled trials comparing DOACs with VKA), no statistical significant differences were found between treatment groups.^35^, ^36^ Cheung et al. observed a lower PTS prevalence in patients treated with rivaroxaban (29%) compared with warfarin (40%) 5 years after index DVT in the Einstein DVT study (*n* = 336) (hazard ratio 0.8, 95% CI 0.5–1.1).^35^ QoL was not measured in this study. Wik et al. assessed PTS according to the patient‐reported Villalta score in the DABI‐PTS study (*n* = 253).^36^ Nine years after index DVT in the RE‐COVER study, PTS was reported by 63% of patients with DVT treated with dabigatran and by 60% of patients treated with warfarin (crude odds ratio 1.1, 95% CI 0.6–1.8). QoL scores did not differ between treatment groups. Like in our study, the samples of included patients in the follow‐up studies compared with the total number of patients included in the randomized clinical trial were relatively small.

Several other studies have assessed the PTS rate in DVT patients treated with DOACs and VKA.^28^, ^29^, ^30^, ^31^, ^32^, ^33^, ^34^ Rivaroxaban was the investigated drug in the majority of studies. A systematic review and meta‐analysis evaluating seven comparative studies including 2364 DVT patients (833 treated with rivaroxaban) found that rivaroxaban treatment was associated with a lower risk of PTS (pooled unadjusted OR 0.5, 95% CI 0.4–0.7; pooled adjusted OR 0.4, 95% CI 0.4–0.6).^49^ It has to be mentioned that predominantly nonrandomized cohort studies were included in this meta‐analysis and that older historical cohorts were used as control in two studies.^31^, ^33^ Furthermore, follow‐up duration often was <5 years and varied between treatment arms. Last, INR was not always well‐controlled in patients using VKA. QoL was studied in only one prospective cohort of 309 DVT patients: mean VEINES‐QOL score was 52 in patients treated with rivaroxaban and 48 in patients treated with warfarin, 22 and 27 months after index event,^30^ which is comparable to our results on QoL.

There are several potential explanations for our findings. Because our study population consisted of a subgroup of the original trial population, confounding owing to between‐group differences could have obscured an association between treatment and PTS. To assess the robustness of the association we decided to adjust for possible (available) confounders in different models; one model for which confounders were selected based on clinical reasoning and one model for which confounders were selected based on *p* values. Both models showed an adjusted OR of 1.6; one was statistically significant, whereas the other was not. It is possible that in a well‐controlled clinical trial setting in which patients adhered to protocol that included drug accountability, INRs were more often in the therapeutic range than they would have been in clinical practice, and advantages of the stable anticoagulant effect of edoxaban over warfarin may be obscured. We also evaluated disease‐specific and generic health‐related QoL in this study. Even though we did find a trend for a higher frequency of PTS in the edoxaban treatment arm, we did not observe clinically relevant differences in disease specific or in generic health‐related QoL between groups. This may be explained as a larger proportion of moderate PTS drove the observed PTS difference, whereas the proportion of severe PTS was similar in both groups.

Strengths of our study are the randomized, blinded design of the treatment of acute VTE and the thorough assessment of different health aspects including original Villalta score assessed by trained physicians and both disease‐specific and generic health‐related QoL assessment. However, we acknowledge that the small sample of included patients in this follow‐up study is the most important limitation leading to potential selection bias. Selection bias might cause both over‐ and underestimation of the prevalence of PTS. Especially after 7 years since randomization, patients with PTS, leg symptoms, and/or other comorbidities would be more likely to have up‐to‐date contact details in their medical charts and might be more willing to visit the hospital. This might also explain the somewhat high prevalence of PTS as compared with 20%–50% reported in literature^1^ and the percentages reported in a study with shorter duration of follow‐up.^35^ On the other hand, patients with extensive DVT might have suffered from underlying disease that was, at the time of randomization in the Hokusai‐VTE trial still unknown, and are therefore not represented in our study population. This could explain the lower proportion of common femoral and iliac DVT in our sample compared with the original Hokusai‐VTE trial. Differences between our sample and the Hokusai‐VTE trial population might lead to limitations in generalizability. Moreover, no information on treatment allocation of patients who declined participation was available and we were unable to account for the competing risk bias of mortality, which poses a risk for attrition bias. Furthermore, because of the observational nature of this follow‐up study, unmeasured confounding (e.g,. history of ipsilateral DVT or a Villalta score at baseline) could have affected the findings. We attempted to control for confounding by adjusting for a wide range of potential confounders and explored the associations by means of two different multivariable models. However, unmeasured confounding cannot be fully excluded. In addition, we evaluated the association between the occurrence of PTS and treatment group by means of logistic regression analyses. Last, the high drug adherence rates in this study could limit the generalizability of our results for daily clinical practice.

Clinical implications of our findings are limited. The lack of difference in PTS prevalence and QoL outcomes between treatment arms indicates that DOAC therapy does not necessarily improve long‐term sequelae compared with well‐managed VKA therapy. PTS remains a common complication after DVT, regardless of type of anticoagulant used to treat DVT, and our study suggests that choosing edoxaban over warfarin to treat acute DVT should not be based on long‐term outcomes. Future studies should focus on assessing PTS prevalences in patients included in clinical practice data studies with associated drug adherence rates, such as data from the ETNA‐VTE‐Europa study.^50^

## 5 CONCLUSION

Contrary to our hypothesis, the observed prevalence of PTS tended to be higher in patients with acute DVT treated with edoxaban compared with warfarin. Health‐related QoL did not differ between treatment groups. Further research is warranted to unravel the role of anticoagulant therapy on thrombus resolution and development of PTS.

## AUTHOR CONTRIBUTIONS

The steering committee (I.M. Bistervels, R. Bavalia, J. Beyer‐Westendorf, P. Verhamme, S. Middeldorp [executives], and A.J. ten Cate‐Hoek, S.R. Kahn, and I. Quéré) designed the study. All Hokusai PTS investigators recruited patients and collected data. B.A. Hutten, M. Shi, Y. Ling, I.M. Bistervels, and R. Bavalia contributed to the data analysis. I.M. Bistervels and R. Bavalia wrote the first draft of the manuscript and all authors critically reviewed and revised the manuscript. The final manuscript was approved by all authors.

## RELATIONSHIP DISCLOSURE

J.B.W. reports personal fees and other from Bayer HealthCare, personal fees and other from Boehringer. Ingelheim, personal fees and other from BMS/Pfizer, personal fees and other from CSL Behring, personal fees and other personal fees and other from Daiichi Sankyo, personal fees and other from LEO Pharma, outside the submitted work. F.C. reports grants from BMS/Pfizer, personal fees and other from Bayer HealthCare, personal fees and other from AstraZeneka, personal fees and other from MSD, personal fees and other from GSK, other from Janssen, personal fees and other from Novartis, outside the submitted work. M.C. reports personal fees from Bayer HealthCare, personal fees from Boehringer Ingelheim, personal fees from Bristol‐Myers Squib, personal fees from CSL Behring, personal fees from Daiichi Sankyo, personal fees from Pfizer, personal fees from Portola, personal fees from Sanquin Blood Supply, outside the submitted work. W.G. reports grants and other from Bayer HealthCare, grants and other from Pfizer, other from Novartis, other from Amgen, other from Principia, from Sanofi, other from MSD, other from Sobi, outside the submitted work. K. Meijer receives other from Bayer HealthCare, other from Uniqure, other from Alexion, other from Octapharma, outside the submitted work. S.M. reports grants from GSK, grants from BMS/Pfizer, grants from Aspen, grants from Daiichi Sankyo, grants from Bayer HealthCare, grants from Boehringer Ingelheim, grants from Sanofi, grants from Portola, outside the submitted work. M.A.S.P. reports honoraria from Bayer, Pfizer, and Leo Pharma. O.S. reports grants from Daiichi‐Sankyo, during the conduct of the study; grants, personal fees, and nonfinancial support from Bayer HealthCare, grants, personal fees and nonfinancial support from BMS, personal fees and non‐financial support from Pfizer, grants, personal fees and non‐financial support from Boehringer Ingelheim, grants and personal fees from MSD, personal fees from Chiesi, grants and personal fees from Boston Scientifics, outside the submitted work. S.M.S. reports receiving consulting fees from Bayer and Boehringer Ingelheim, and lecture fees from Bayer, Boehringer Ingelheim, and Bristol‐Myers Squibb–Pfizer. P.V. reports grants and personal fees from Bayer HealthCare, grants and personal fees from Boehringer Ingelheim, grants and personal fees from BMS/Pfizer, personal fees from Daiichi Sankyo, personal fees from LEO Pharma, personal fees from Anthos therapeutics, personal fees from Portola Pharmaceuticals/Alexion, outside the submitted work. M.G., M.S., and Y.L. report being an employee of Daiichi‐Sankyo. No other potential conflict of interest with relation to this study were reported.

## Supporting information


Appendix S1

Figure S1
