# Supplementary material for: Postthrombotic syndrome and quality of life after deep vein thrombosis in patients treated with edoxaban versus warfarin
Source: Res Pract Thromb Haemost. 2022 Jul 1;6(5):e12748. doi: 10.1002/rth2.12748 (PMC9248314; doi:10.1002/rth2.12748)
Supplement: Supplementary file 1 — Appendix S1 [file RTH2-6-e12748-s001.docx]

## Supplementary Appendix

**Steering Committee**

Executives:

S. Middeldorp (chair)

R. Bavalia

J. Beyer-Westendorf

I.M. Bistervels

P. Verhamme

Other members:

A.J. ten Cate-Hoek

S.R. Kahn

I. Quéré

**Daiichi Sankyo Clinical Team & Statistical Support**

M. Grosso

Y. Lin

M. Shi

**Study centers:**

*Australia* – (7 patients, 2 centers)

P. Carroll, Redcliffe (3)

A. Hugman, Sydney (4)

*Belgium* – (9 patients, 1 center)

P. Verhamme, Leuven (9)

*Canada* – (34 patients, 2 centers)

S.R. Kahn, Montreal (8)

M.J. Kovacs, A. Lazo-Langner, J.A. Kovacs, London, Ontario (26)

*France* – (78 patients, 11 centers)

D. Brisot, Castelnau le Lez (9)

F. Couturaud, C. Hoffmann, Brest (13)

N. Falvo, M. Jandot, F. Ghiringhelli, Dijon (26)

E. Ferrari, Nice (2)

K. Montaclair, Le Mans (6)

G. Pernod, Grenoble (2)

I. Quéré, Montpellier (1)

P.M. Roy, Angers (1)

O. Sanchez, Paris (2)

M.-A. Sevestre-Pietri, Amiens (1)

D. Stephan, M. Cordeanu, Strasbourg (15)

*Germany* – (98 patients, 2 centers)

J. Beyer-Westendorf, C. Naue, L. Tittl, C. Köhler, S. Marten, H. Mizera, Dresden (57);

S.M. Schellong, R.Frommhold, B. Voigts, M. Brudzinski, M. Hahn, Dresden (41).

*Italy* – (1 patient, 1 center)

M. Cattaneo, Milan (1).

*The Netherlands* – (80 patients, 5 centers)

W.G. Boersma, L. Oudeman, Alkmaar (15)

A.J. ten Cate-Hoek, Maastricht (7)

K. Meijer, A.B.U. Mäkelburg, Groningen (19)

S. Middeldorp, R. Bavalia, I.M. Bistervels, Amsterdam (25)

M. ten Wolde, I.M. Bistervels, Almere (14)

*Norway* – (9 patients, 2 centers)

W. Ghanima, Østfold (8)

H.S. Wik, Oslo (1)

**Supplementary Figure S1.** Time line of Hokusai-VTE trial and Hokusai PTS study

**
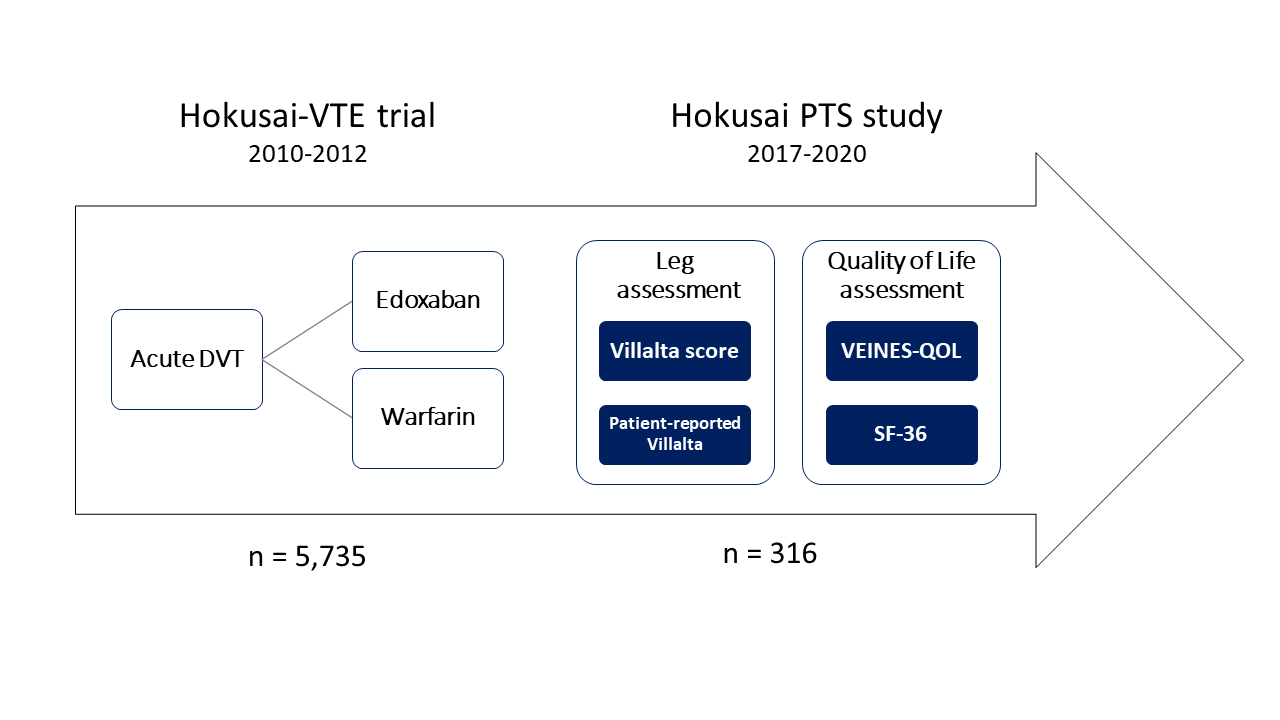
**

**Supplementary Table S1.** Overview patient and thrombus characteristics at baseline of patients included in the Hokusai-VTE trial with deep vein thrombosis

|  | **All patients with DVT**  **in Hokusai-VTE trial**  **(n = 5,735)** | | **Patients from study sites not invited for participation in Hokusai PTS study**  **(n=4,205)** | **Patients from study sites invited for participation in Hokusai PTS study (n=1,530)** | **Patients who participated in Hokusai PTS study (n=316)** |
| --- | --- | --- | --- | --- | --- |
|  | **Edoxaban* (n=2,878)** | **Warfarin* (n=2,857)** |  |  |  |
| Mean age in years (SD) | 54.9 (15.9) | 55.1 (15.8) | 54.6 (15.9) | 56.1 (16.0) | 55.5 (14.5) |
| Male sex – n (%) | 1747 (60.7) | 1725 (60.4) | 2565 (61.0) | 907 (59.3) | 171 (54.1) |
| Mean weight in kg (SD) | 81.7 (19.3) | 82.6 (19.7) | 80.1 (18.8) | 88.0 (20.2) | 85.1 (18.3) |
| Mean BMI in kg/m^2^ (SD) | 28.0 (5.5) | 28.3 (5.9) | 27.8 (5.5) | 29.2 (6.1) | 28.2 (5.1) |
| Thrombus location (most proximal site) – n (%)  Popliteal vein  Superficial femoral vein  Common femoral or iliac vein  Unknown | 752 (26.1)  941 (32.7)  1142 (39.7)  43 (1.5) | 736 (25.8)  916 (32.1)  1170 (41.0)  35 (1.2) | 980 (23.3)  1303 (31.0)  1869 (44.4)  53 (1.3) | 508 (33.2)  554 (36.2)  443 (29.0)  25 (1.6) | 124 (39.2)  113 (35.8)  74 (23.4)  5 (1.6) |
| Median duration of anticoagulant treatment in months (SD) | 9.3 (6.0) | 9.3 (6.0) | 10.7 (6) | 6.4 (5.9) | 8.0 (6.0) |
| Patients receiving 30 mg of Edoxaban at randomization † - n (%) | 493 (17.1) | 471 (16.5) | 805 (19.1) | 159 (10.4) | 23 (13.7) |
| Patients who died during the Hokusai-VTE trial – n (%) | 90 (3.1) | 89 (3.1) | 144 (3.4) | 35 (2.3) | Not applicable |

**LEGEND TABLE S1.** *Abbreviations: SD: standard deviation, n: number, kg: kilogram*

** Both edoxaban and warfarin treatment were preceded by enoxaparin.*

*† Patients with a body weight below 60 kg or a creatinine clearance of 30 to 50 ml per minute, as well as patients who were receiving concomitant P-glycoprotein inhibitors such as verapamil or quinidine, received 30 mg instead of 60 mg of edoxaban to maintain similar exposure to the cohort receiving 60 mg.*

**Supplementary Table S2.** Overview of the adjusted variables in the model 1 † and 2 ǂ for the PTS outcomes

|  | **OUTCOMES** | | |
| --- | --- | --- | --- |
| **Model 1: based on clinical reasoning** † | PTS § | Severe PTS* | Venous Ulcer |
| **Age** at inclusion Hokusai PTS study |  |  |  |
| **Male sex** | X |  |  |
| **BMI** at inclusion Hokusai PTS study | X | X |  |
| **≥ 2 VTE in history** at inclusion Hokusai PTS study |  | X | X |
| **Cardiovascular disease** at inclusion Hokusai PTS study |  | X |  |
| **Any concomitant medication** at inclusion Hokusai PTS study |  |  |  |
| **Awareness of randomized treatment** in Hokusai-VTE trial |  |  |  |
| **Thrombus location index DVT**  (popliteal, superficial femoral, common femoral or iliac vein) | X | X |  |
| **Duration of anticoagulant treatment** in Hokusai-VTE trial | X |  |  |
| **Model 2: based on p-values** ǂ | PTS | Severe PTS | Venous Ulcer |
| **Male sex** | X |  |  |
| **Cardiovascular disease** at inclusion Hokusai PTS study |  | X |  |
| **Chronic analgesic use** at inclusion Hokusai PTS study | X |  |  |
| **Use of concomitant antiplatelet therapy** in Hokusai-VTE trial |  | X | X |

**LEGEND TABLE S2.**

*Abbreviations: PTS: post-thrombotic syndrome, BMI: body mass index, VTE: venous thromboembolism, DVT: deep vein thrombosis*

*† adjusted by variables from model 1 (clinical reasoning) as described in the methods section*

*ǂ adjusted by variables derived by model 2 (p-value<0.25) as described in the methods section.*

*§ According to ISTH scoring: Villalta score of 5 or higher or presence of venous ulcer, at least 6 months after DVT*

** Villalta score of 15 or more or presence of a venous ulcer*

**Supplementary Table S3.** Crude and adjusted mean differences for generic health-related quality of life for patients with index DVT

| **SF-36 domain – mean (SD)** | **Edoxaban***  **(n=168)** | **Warfarin***  **(n=148)** | **Crude mean difference**  **(95%CI)** | **Adjusted mean difference**  **(95%CI)** † | **Adjusted mean difference**  **(95%CI)** ǂ |
| --- | --- | --- | --- | --- | --- |
| SF-36 Physical Functioning | 75.0 (26.8) | 78.5 (22.3) | -3.5 (-9.1 to 2.1) | -5.6 (-10.7 to -0.6) | -5.7 (-10.5 to -0.9 |
| SF-36 Social Functioning | 83.5 (23.5) | 85.0 (21.3) | -1.5 (-6.5 to 3.6) | -3.8 (-8.7 to 1.1) | -2.4 (-7.1 to 2.4) |
| SF-36 Role Physical | 71.1 (39.4) | 73.9 (39.1) | - 2.8 (-11.7 to 6.0) | -7.7 (-16.2 to 0.9) | -6.2 (-14.3 to 1.8) |
| SF-36 Role emotional | 82.8 (34.6) | 79.3 (34.9) | 3.6 (-4.3 to 11.4) | 0.2 (-7.6 to 8.0) | 2.1 (-5.6 to 9.9) |
| SF-36 Mental Health | 76.5 (18.5) | 76.8 (19.3) | -0.3 (-4.6 to 3.9) | -2.7 (-6.8 to 1.3) | -1.1 (-5.2 to 3.0) |
| SF-36 Vitality | 64.4 (20.0) | 60.4 (20.5) | 4.0 (-0.6 to 8.6) | 1.5 (-2.8 to 5.8) | 2.1 (-2.2 to 6.4) |
| SF-36 Bodily Pain | 72.1`(25.8) | 71.1 (27.7) | 1.0 (-5.0 to 7.1) | -3.1 (-8.7 to 2.6) | -1.8 (-7.3 to 3.7) |
| SF-36 General Health perceptions | 65.6 (19.8) | 66.1 (20.4) | -0.5 (-5.0 to 4.1) | -2.8 (-7.2 to 1.6) | -1.7 (-6.0 to 2.7) |

**LEGEND TABLE S3.**

*Abbreviations: SF-36: Short Form 36 items, SD: standard deviation, n: number, 95%CI: 95% confidence interval.*

** Both edoxaban and warfarin treatment were preceded by enoxaparin.*

*† adjusted by variables from model 1 (clinical reasoning) as described in the methods section.*

*ǂ adjusted by variables derived by model 2 (p<0.25) as described in the methods section. Details on the included variables per model are presented in the supplementary appendix. A negative mean difference implies a difference in favor of warfarin, a positive mean difference implies a difference in favor of edoxaban.*

**Supplementary Table S4.** Overview of the adjusted variables in the model 1 † and 2 ǂ for the quality of life outcomes (VEINES-QOL and all domains of SF-36)

|  |  | **OUTCOMES** | | | | | | | | |  | |  | | |
| --- | --- | --- | --- | --- | --- | --- | --- | --- | --- | --- | --- | --- | --- | --- | --- |
| **Model 1: based on clinical reasoning** † | VEINESQOL | | PF | SF | RP | RE | MH | VT | BP | GH | | PCS | | MCS |  |
| **Age**  at inclusion Hokusai PTS study |  | | X |  | X | X | X | X | X | X | | X | |  |  |
| **Male sex** | X | | X |  | X | X | X | X | X | X | | X | | X |  |
| **BMI**  at inclusion Hokusai PTS study |  | | X | X |  |  | X |  |  |  | |  | | X |  |
| **Cardiovascular disease**  at inclusion Hokusai PTS study |  | |  |  | X |  |  |  |  |  | |  | |  |  |
| **Musculoskeletal disease**  at inclusion Hokusai PTS study | X | | X | X | X |  |  |  | X | X | | X | |  |  |
| **Neurological disease**  at inclusion Hokusai PTS study |  | |  |  |  |  |  |  |  | X | |  | |  |  |
| **Psychiatric disorder**  at inclusion Hokusai PTS study |  | | X | X | X |  | X |  | X | X | | X | | X |  |
| **Chronic analgesic use**  at inclusion Hokusai PTS study | X | | X | X | X | X |  | X | X | X | | X | |  |  |
| **Thrombus location index DVT**  (popliteal, superficial femoral, common femoral or iliac vein) | X | |  | X |  |  |  |  | X | X | | X | |  |  |
| **≥80% compliance to assigned treatment** in Hokusai-VTE trial |  | | X |  | X |  |  |  |  |  | |  | |  |  |
| **Awareness of randomized treatment** in Hokusai-VTE trial |  | |  |  |  |  |  |  |  |  | |  | |  |  |
| **Model 2: based on p-values** ǂ | VEINES QOL | | PF | SF | RP | RE | MH | VT | BP | GH | | PCS | | MCS |  |
| **Male sex** | X | | X | X | X |  | X | X | X | X | | X | | X |  |
| **Cardiovascular disease**  at inclusion Hokusai PTS study |  | | X |  |  |  |  | X |  | X | | X | |  |  |
| **Neurological disease**  at inclusion Hokusai PTS study |  | |  |  |  |  |  |  |  |  | |  | |  |  |
| **Psychiatric disorder**  at inclusion Hokusai PTS study |  | | X | X | X | X | X |  | X | X | | X | | X |  |
| **Chronic analgesic use**  at inclusion Hokusai PTS study | X | | X | X | X | X |  | X | X | X | | X | |  |  |
| **Use of concomitant antiplatelet therapy** in Hokusai-VTE trial |  | | X | X |  |  | X |  | X |  | |  | |  |  |
| **Years since randomization in Hokusai-VTE trial** | X | |  |  |  | X | X | X | X | X | |  | | X |  |

**LEGEND TABLE S4.**

*Abbreviations: BMI: body mass index, DVT: deep vein thrombosis, VTE: venous thromboembolism, PTS: post-thrombotic syndrome, VEINES-QOL: Venous Insufficiency Epidemiological and Economic Study- Quality of Life Questionnaire, SF-36 domains: PF=physical functioning; SF=social functioning; RP=role physical complaints; RE=role emotional complaints; ME=mental health; VI=vitality; BP=bodily pain; GH=general health, PCS=physical component score, MCS=mental component score.*

*† adjusted by variables from model 1 (clinical reasoning) as described in the methods section*

*ǂ adjusted by variables derived by model 2 (p-value<0.25) as described in the methods section*

**S5.** Study outcomes for patients who were unaware of randomization result only (n=217)

|  | **Patients blinded (n=217)** | | |
| --- | --- | --- | --- |
| **Primary outcome – n (%)** | **Edoxaban***  **(n=115)** | **Warfarin***  **(n=102)** | **Crude**  **Odds Ratio**  **(95%CI)** |
| Post-thrombotic syndrome according to ISTH scoring § | 62 (53.9) | 62 (47.8) | 1.6 (0.9 to 2.8) |
| Villalta severity score  Mild PTS (5-9)  Moderate PTS (10-14)  Severe PTS (>14 or leg ulcer) | 42 (67.7)  15 (24.2)  5 (8.1) | 28 (45.2)  11 (17.7)  4 (6.5) | -  -  1.1 (0.3 to 4.3) |
| Ipsilateral leg ulcer | 3 (2.6) | 1 (1.0) | 2.8 (0.3 to 26.4) |
| **Secondary outcome – mean (SD)** | **Edoxaban***  **(n=115)** | **Warfarin***  **(n=102)** | **Crude mean difference**  **(95%CI)** |
| VEINES-QOL | 49.4 (10.0) | 50.0 (10.0) | -0.6 (-3.3 to 2.1) |
| SF-36, PCS | 45.5 (10.5) | 46.5 (9.7) | -1.0 (-3.8 to 1.8) |
| SF-36, MCS | 51.7 (10.1) | 51.5 (9.9) | 0.0 (-2.7 to 2.7) |

**LEGEND TABLE S5.**

*Abbreviations: n: number, 95%CI: 95% confidence interval, PTS: post-thrombotic syndrome, VEINES-QOL: Venous Insufficiency Epidemiological and Economic Study- Quality of Life Questionnaire, SF-36: Short Form 36 items, PCS: physical component score, MCS: mental component score*

** Both edoxaban and warfarin treatment were preceded by enoxaparin.*

*§ Villalta score of 5 or higher or presence of venous ulcer, at least 6 months after DVT*

*Missing values for patients who were blinded only: VEINES-QOL was missing for 3 patients (1 treated with edoxaban, 2 treated with warfarin), SF-36 PCS and MCS was missing for 8 patients (2 treated with edoxaban, 6 treated with warfarin)*.

*Missing values for patients and clinicians who were blinded only: SF-36 PCS and MCS was missing for 2 patients (1 treated with edoxaban, 1 treated with warfarin).*

**Table S6.** Study outcomes in patients who stopped (n=161) and who continued their anticoagulation (n=127) after the end of the Hokusai-VTE trial until inclusion in the Hokusai PTS study

|  | **Patients who stopped anticoagulation (n=161)** | | | **Patients who continued anticoagulation (n=127)** | | |
| --- | --- | --- | --- | --- | --- | --- |
| **Primary outcome – n (%)** | **Edoxaban***  **(n=85)** | **Warfarin***  **(n=76)** | **Crude**  **Odds Ratio**  **(95%CI)** | **Edoxaban***  **(n=65)** | **Warfarin***  **(n=62)** | **Crude**  **Odds Ratio**  **(95%CI)** |
| Post-thrombotic syndrome according to ISTH scoring § | 42 (49.4) | 30 (39.5) | 1.5 (0.8 to 2.8) | 37 (56.9) | 27 (44.3) | 1.7 (0.8 to 3.4) |
| Villalta severity score  Mild PTS (5-9)  Moderate PTS (10-14)  Severe PTS (>14 or leg ulcer) | 31(73.8)  9 (21.4)  2 (4.8) | 17 (56.7)  9 (30.0)  4 (13.3) | -  -  0.4 (01 to 2.4) | 27 (73.0)  4 (10.8)  6 (16.2) | 20 (74.1)  5 (18.5)  2 (7.4) | -  -  3.0 (0.6 to 15.5) |
| Ipsilateral leg ulcer | 2 (2.4) | 1 (1.3) | 1.8 (0.2 to 20.3) | 3 (4.6) | 0 (0.0) | - |
| **Secondary outcome – mean (SD)** | **Edoxaban***  **(n=85)** | **Warfarin***  **(n=76)** | **Crude mean difference**  **(95%CI)** | **Edoxaban***  **(n=65)** | **Warfarin***  **(n=62)** | **Crude mean difference**  **(95%CI)** |
| VEINES-QOL | 49.5 (10.3) | 49.5 (10.9) | 0.0 (-3.4 to 3.3) | 50.1 (9.1) | 49.6 (10.0) | 0.5 (-2.9 to 3.9) |
| SF-36, PCS | 46.3 (10.7) | 46.4 (10.1) | 0.1 (-3.4 to 3.3) | 44.0 (11.1) | 46.9 (10.7) | -2.9 (-6.8 to 1.0) |
| SF-36, MCS | 50.9 (10.5) | 50.6 (9.7) | 0.3 (-3.0 to 3.5) | 54.0 (9.7) | 52.6 (9.9) | 1.4 (-2.0 to 4.8) |

**LEGEND TABLE S6.**

*Abbreviations: no.: number, 95%CI: 95% confidence interval, PTS: post-thrombotic syndrome, VEINES-QOL: Venous Insufficiency Epidemiological and Economic Study- Quality of Life Questionnaire, SF-36: Short Form 36 items, PCS: physical component score, MCS: mental component score*

** Both edoxaban and warfarin treatment were preceded by enoxaparin.*

*§ Villalta score of 5 or higher or presence of venous ulcer, at least 6 months after DVT*

*Missing values for patient who stopped anticoagulation: VEINES-QOL was missing for 3 patients (1 treated with edoxaban, 2 treated with warfarin), SF-36 PCS and MCS was missing for 7 patients (3 treated with edoxaban, 4 treated with warfarin)*

*Missing values for patients who continued anticoagulation: Villalta score was not assessed in 1 patient treated with warfarin, SF-36 PCS and MCS was missing for 4 patients (1 treated with edoxaban, 3 treated with warfarin)*
