# Supplementary figures and images for: Postthrombotic syndrome and quality of life after deep vein thrombosis in patients treated with edoxaban versus warfarin
Source: Res Pract Thromb Haemost. 2022 Jul 1;6(5):e12748. doi: 10.1002/rth2.12748 (PMC9248314; doi:10.1002/rth2.12748)

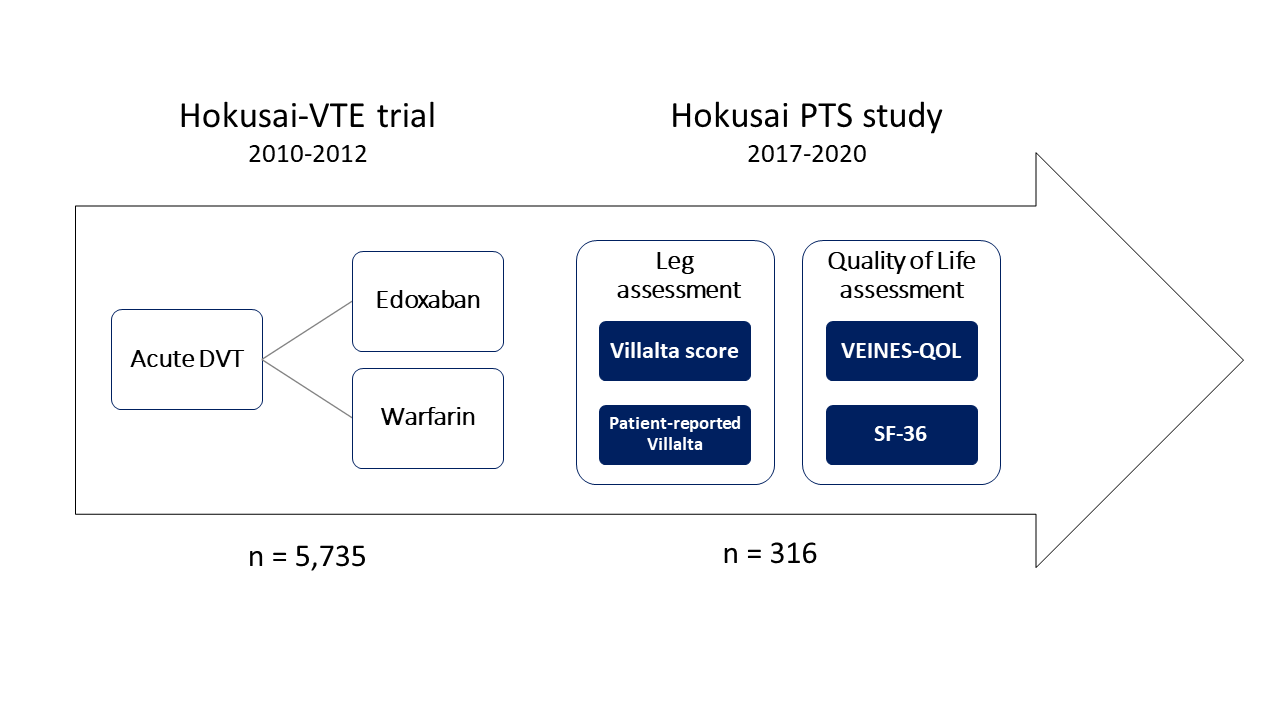

Supplement: Supplementary file 2 — Figure S1 [file RTH2-6-e12748-s002.tif]
